# A Fenclorim Molecularly Imprinted Electrochemical Sensor Based on a Polycatechol/Ti_3_C_2_T_x_ Composite

**DOI:** 10.3390/s25185838

**Published:** 2025-09-18

**Authors:** Xiu Liu, Xing Tang, Hongjun Chen, Xiang Wu, Zitong Fu, Mingyu Peng, Chenzhong Jin, Jun Guo

**Affiliations:** 1 Key Laboratory of Green Control of Crop Pests in Hunan Higher Education, Hunan Provincial Collaborative Innovation Center for Field Weeds Control, Hunan University of Humanities, Science and Technology, Loudi 417000, China; liuxiu841027@163.com (X.L.); xingtangrw@163.com (X.T.); jinchenzhongrw@163.com (C.J.); 2 Hunan Provincial Key Laboratory of Fine Ceramics and Powder Materials, School of Materials and Environmental Engineering, Hunan University of Humanities, Science and Technology, Loudi 417000, China; xiangwu0827@163.com (X.W.); zitongfu2024@163.com (Z.F.); pengmingyu05@163.com (M.P.); renwenguojun@126.com (J.G.)

**Keywords:** electrochemical sensor, molecularly imprinted polymers, titanium carbide nanomaterials, fenclorim, herbicide safener

## Abstract

Given the significance of safeners and their potential to emit harmful substances into the environment, it is essential to develop suitable analytical methods for detecting these compounds. This study presents a molecularly imprinted electrochemical sensor designed for the sensitive and rapid detection of fenclorim (FM), a type of safener. Titanium carbide nanomaterials (Ti_3_C_2_T_x_) were electrochemically deposited onto the glassy carbon electrode (GCE) to enhance electron transfer. Subsequently, molecularly imprinted polymers were fabricated through the electropolymerization of catechol in the presence of FM. The electrochemical behavior of each modified electrode was investigated using differential pulse voltammetry (DPV) and electrochemical impedance spectroscopy (EIS). Under optimized experimental conditions, the MIP/Ti_3_C_2_T_x_/GCE sensor demonstrated a linear relationship with FM concentration ranging from 5 to 300 nM, with a limit of detection (LOD) of 1.56 nM (S/N = 3). Additionally, the sensor demonstrated excellent selectivity, stability, and reproducibility for FM detection and was successfully utilized for quantifying FM in real water samples.

## 1. Introduction

Herbicide safeners are co-applied with herbicides to protect crops from herbicide toxicity. Dichloroacetamide safeners (such as fenclorim, AD-67, benoxacor, dichlormid, and furilazole) are typically formulated with chloroacetamide herbicides such as pretilachlor, acetochlor, and metolachlor, mainly to protect crops (rice and soybeans) [1,2,3]. Both herbicides and safeners have been detected in the environment, including soil, streams, and surface water, and their concentrations tend to rise in tandem [3,4,5]. When herbicides are detected in streams, safeners are typically present as well, albeit at significantly lower levels. For example, the four most common dichloroacetamide safeners have been detected in surface waters across the Midwestern U.S., highlighting the need for further investigation into their environmental fate [2,3,6,7,8]. Although safeners are classified as “inert” ingredients, they are biologically active and exhibit low-to-moderate toxicity to aquatic organisms (LC_50_ values for freshwater fish range from 1.4 to 4.6 mg/L) [2,7]. Notably, some safeners, including furilazole and AD-67, are carcinogenic and mutagenic [7,9,10]. Furthermore, when safeners interact with non-target aquatic organisms and microorganisms, they may form transformation products with enhanced biological activity or toxicity [7,8,9,10]. In iron-reducing environments, dichloroacetamide safeners can even generate herbicidally active derivatives [7]. Considering the significance of these safeners and their potential to generate harmful substances in the environment, it is essential to develop suitable analytical methods for their detection, thereby supporting environmental risk assessment and management.

Currently, high-resolution mass spectrometry (HRMS) coupled with gas chromatography (GC) or high-performance liquid chromatography (HPLC) is commonly used for the analysis of safeners and pesticides [11,12,13]. Additional approaches, such as matrix-assisted laser desorption/ionization mass spectrometry imaging and radio-HPLC, have also been employed [7,10,14]. However, these methods are often complex, costly, and time-consuming, requiring sophisticated instrumentation and elaborate extraction steps. By contrast, electrochemical sensing has emerged as a promising alternative, offering ease of use, a rapid response, cost-effectiveness, and adequate sensitivity [15,16,17,18]. For effective detection of safeners using electrochemical sensors, high selectivity is crucial to minimize interference from herbicides and structural analogs. Various recognition mechanisms, including antigen–antibody interactions, the biotin–avidin system, and molecularly imprinted polymers (MIPs), have been used to construct highly selective electrochemical sensors [18,19]. Among these strategies, the molecular imprinting technique is exceptionally versatile for fabricating selective sensors. While many molecularly imprinted electrochemical sensors have been developed for pesticides, herbicides, fungicides, insecticides, and other toxic organics [15,16,20,21], relatively few studies have targeted herbicide safeners, especially dichloroacetamide safeners.

Fenclorim has demonstrated acute inhalation toxicity in humans and persistent adverse effects on aquatic ecosystems [12]. Existing research has mainly explored the uptake, persistence, translocation, and metabolism of FM in treated rice plants, as well as its environmental hazards [12]. Given the increasing concerns about FM residues, there is a pressing need to develop rapid, selective, and sensitive analytical methods for detecting and quantifying FM residues. The objective of this study is to develop a molecularly imprinted electrochemical sensor for the detection of dichloroacetamide safeners. Fenclorim, a common safener in commercial herbicide formulations, was selected as the target molecule for imprinting. Briefly, layered titanium carbide nanomaterials (Ti_3_C_2_T_x_) were electrochemically deposited on a glassy carbon electrode (GCE) to enhance electron transfer. Subsequently, molecularly imprinted polymers were formed by electropolymerizing catechol in the presence of FM. After the removal of template molecules, an FM MIP-based electrochemical sensor modified with Ti_3_C_2_T_x_ (MIP/Ti_3_C_2_T_x_/GCE) was fabricated.

## 2. Materials and Methods

### 2.1. Materials and Reagents

Titanium aluminum carbide (Ti_3_C_2_T_x_), hydrofluoric acid (HF), tetramethyl ammonium hydroxide (TMAOH, 25% aqueous solution), CH_3_COOH, CH_3_COONa, ethanol, methanol, acetonitrile, acetone, catechol, potassium hexacyanoferrate(II), potassium hexacyanoferrate(III), potassium chloride (KCl), fenclorim (FM), pretilachlor, and benoxacor were purchased from Aladdin Chemistry Co., Ltd. (Shanghai, China). Fenclorim was dissolved in acetone to prepare a 100 mM stock solution. Deionized water (Millipore Milli-Q, 18.2 MΩ·cm) was used to prepare the solutions. All reagents were of analytical grade and used without further purification.

### 2.2. Apparatus

A field-emission scanning electron microscope (SEM; JEOL-JSM-7600F, JEOL Ltd., Tokyo, Japan) was applied to characterize the surface morphology of the modified electrodes. All electrochemical measurements, including cyclic voltammetry (CV), differential pulse voltammetry (DPV), and electrochemical impedance spectroscopy (EIS), were performed using a CHI 660B electrochemical workstation adopting a three-electrode system. Specifically, the Pt plate (1.0 × 1.0 cm^2^) served as the counter-electrode, a saturated Ag/AgCl electrode acted as the reference electrode, and the prepared modified glassy carbon electrode (GCE) functioned as the working electrode. The glassy carbon electrode (3.0 mm in diameter), reference electrode, and counter-electrode were all purchased from Shanghai Chenhua Technology Co., Ltd. (Shanghai, China). All of the DPV measurements were conducted within a potential range of −0.2 V to 0.6 V at a scanning rate of 50 mV/s, using 20 mL of 0.1 M KCl supporting electrolyte containing 5.0 mM of [Fe(CN)_6_]^3−/4−^ (1:1) redox probe. The general parameters for the EIS assessment were set as follows: the signal amplitude was set to 5.0 mV, and the frequency range was adjusted to 0.01–100,000 Hz, conducted in 20 mL of 0.1 M KCl supporting electrolyte containing 5.0 mM of [Fe(CN)_6_]^3−/4−^. The equivalent electrical circuit was simulated using ZSim Demo software (version 3.30d). A Thermo Sorvall Biofuge Stratos centrifuge (Electron LED GmbH, Osterode, Germany) was used for centrifugation.

### 2.3. Synthesis of Layered Titanium Carbide (Ti_3_C_2_T_x_)

Layered titanium carbide (Ti_3_C_2_T_x_) was prepared using the acid etching method with modifications according to previous reports [22,23]. Initially, 2.0 g of Ti_3_AlC_2_ powder was dispersed in 16 mL of 10% HF in a 50 mL plastic beaker and stirred continuously for 24 h at room temperature. The plastic beaker was covered with a plastic film with small holes, and the entire experiment was conducted in a fume hood. (Caution: HF is a highly corrosive acid and one must always be aware of the risk involved and follow safety protocols when handling HF and HF-producing chemicals.) Then the resultant mixture was centrifugally washed with water until the pH value reached 6.0, and the resulting sediment was collected and dried under vacuum for further use. Following this, 0.5 g of the aforementioned powder was mixed with 10 mL of TMAOH solution and stirred at room temperature for 24 h. After this, the resulting colloidal solution was centrifuged at 1465 g to separate the non-etched crude product, and subsequently centrifuged at 7656 g to collect the required sediment. Subsequently, the Ti_3_C_2_T_x_ sediment was obtained and washed three times with ethanol and water to eliminate any remaining TMAOH. Lastly, the Ti_3_C_2_T_x_ product was dried under vacuum for subsequent use.

### 2.4. Electrochemical Preparation of Ti_3_C_2_T_x_-Modified Electrode (Ti_3_C_2_T_x_/GCE)

Ti_3_C_2_T_x_ was electrochemically deposited onto the electrodes following a previously reported method with slight modifications [24]. Firstly, the bare GCE was carefully polished with 0.03 and 0.01 µm alumina powder slurry and immersed in water and ethanol successively for ultrasonic cleaning, and then dried in a N_2_ stream. Secondly, 20 mL acetate buffer (0.1 M, pH = 5.5) containing Ti_3_C_2_T_x_ (3.0 mg/mL) was sonicated for 30 min to prepare the deposition electrolyte. Subsequently, CV was performed over a potential range of +0.2 V to −1.3 V at a scan rate of 100 mV/s for three cycles. Finally, the resulting Ti_3_C_2_T_x_-modified electrode (Ti_3_C_2_T_x_/GCE) was rinsed with water and then dried at room temperature for further applications.

To evaluate the effects of the number of scanning cycles and scan rate on the electrochemical performance of the Ti_3_C_2_T_x_/GCE during CV deposition, differential pulse voltammetry (DPV) was utilized. Initially, the influence of the number of CV scanning cycles was investigated by conducting CV within the +0.2 V to −1.3 V potential range at a fixed scan rate of 100 mV/s for specific cycle numbers (1, 3, 5, 7, and 10 cycles). The resulting electrodes were then washed with water and underwent DPV measurements. The difference in DPV peak current (ΔI = I − I_0_) before and after deposition was calculated to determine the optimal number of scanning cycles. Once optimal scanning cycles were established, the effect of scan rate was examined by performing CV within the same potential range at various scan rates (50, 75, 100, 125, and 200 mV/s) over three specified cycles. The electrodes were again washed with water and characterized using DPV.

### 2.5. Fabrication Processes of the MIP and NIP Electrodes

**Figure 1:**
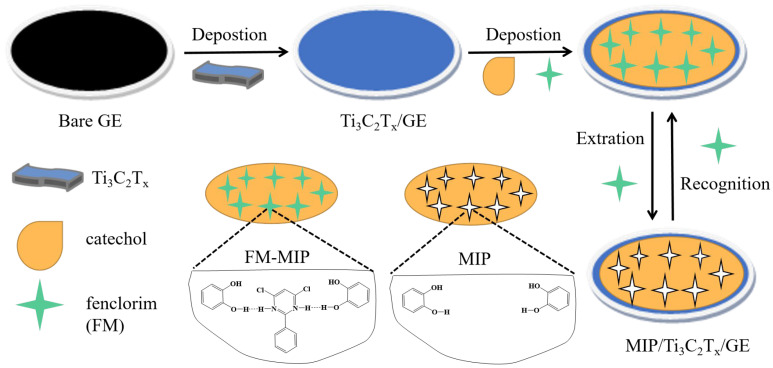
Schematic representation of the preparation of MIP/Ti_3_C_2_T_x_/GCE.

The general fabrication procedure for the fenclorim (FM) molecularly imprinted electrode (MIP/Ti_3_C_2_T_x_/GCE) is presented in Figure 1. Briefly, the Ti_3_C_2_T_x_/GCE was initially immersed in 10 mL acetate buffer (0.1 M, pH 5.0) containing the template FM (1 mM) and the catechol functional monomers (12 mM) for 30 s, and then, polymerization was performed by CV between −0.2 and 0.8 V for 10 cycles at a 75 mV/s scan rate. After electropolymerization, the resultant electrode was immersed in 15 mL methanol–acetonitrile (1:2, *v*/*v*) solution and stirred for 30 min to remove the embedded template molecules, and subsequently rinsed with water and dried. For comparison, the non-imprinted polymer (NIP) was prepared using the same method but without adding the target template molecules, and the obtained electrode was denoted as NIP/Ti_3_C_2_T_x_/GCE.

### 2.6. Optimization of Parameters for the Developed MIP/Ti_3_C_2_T_x_/GCE Sensor

To evaluate the effects of the number of CV scanning cycles, template-to-monomer ratios, and incubation time on the electrochemical performance of the MIP/Ti_3_C_2_T_x_/GCE sensor, differential pulse voltammetry (DPV) was utilized. Initially, the influence of the number of CV scanning cycles was investigated by conducting CV within the −0.2 V to +0.8 V potential range at a fixed scan rate of 75 mV/s across specific cycle numbers (5, 10, 15, and 25 cycles) in 10 mL acetate buffer (0.1 M, pH 5.0) containing the template FM (1 mM) and catechol functional monomers (12 mM). The resulting electrodes were washed with water, and then incubated in 10 mL of acetate buffer (0.1 M, pH 7.0) containing 1.0 mM of FM for one hour. Following this, the electrodes were washed again with water and underwent DPV measurements. The difference in the DPV peak currents (ΔI = I − I_0_) before and after CV deposition was calculated to determine the optimal number of scanning cycles. After establishing the optimal CV scanning cycles, the effect of template-to-monomer ratios was examined by performing CV within the same potential range at a scan rate of 75 mV/s over 10 specified cycles in 10 mL acetate buffer (0.1 M, pH 5.0) using varying template-to-monomer ratios (1:5, 1:8, 1:10, 1:12, 1:15), with the concentration of FM fixed at 1.0 mM. The electrodes were washed with water once again, incubated in 10 mL of acetate buffer (0.1 M, pH 7.0) containing 1.0 mM FM for one hour, washed again, and characterized using DPV. Once optimal template-to-monomer ratios were established, the effect of incubation time was examined by performing CV within the same potential range at a scan rate of 75 mV/s over 10 specified cycles in 10 mL acetate buffer (0.1 M, pH 5.0) with a template-to-monomer ratio of 1:12, while maintaining the concentration of FM at 1.0 mM. The electrodes were again washed with water, incubated in 10 mL of acetate buffer (0.1 M, pH 7.0) containing 1.0 mM of FM for various durations (10, 20, 30, 40, 50, and 60 min), washed one final time with water, and characterized using DPV.

### 2.7. Analytical-Performance Measurements

For the investigation of analytical performance, the newly developed MIP/Ti_3_C_2_T_x_/GCE and NIP/Ti_3_C_2_T_x_/GCE were immersed in 10 mL of acetate buffer (0.1 M, pH 7.0) with varying concentrations of FM (0, 5, 10, 25, 50, 100, 200, 300, and 400 nM) for 30 min. After immersion, the electrodes were rinsed with water and analyzed using the DPV method. For selective-recognition tests, MIP/Ti_3_C_2_T_x_/GCE and NIP/Ti_3_C_2_T_x_/GCE were first incubated in 10 mL of acetate buffer (0.1 M, pH 7.0) containing either 100 nM of FM or interfering substances (1.0 mM of benoxacor or 1.0 mM of pretilachlor) for 30 min under continuous stirring. Following this, the electrodes were rinsed with water and then characterized using DPV. For the stability assessments, the newly developed MIP/Ti_3_C_2_T_x_/GCE was incubated in 10 mL of acetate buffer (0.1 M, pH 7.0) containing 50 nM of FM for 30 min. After incubation, the electrodes were washed with water and characterized using DPV. Subsequently, the electrodes were washed again with water and immersed in 15 mL methanol–acetonitrile (1:2, *v*/*v*) solution for 30 min to remove rebound FM. Tests were then conducted every two days according to the above procedures until the 15th day.

### 2.8. Detection of FM in the Actual Samples

Different kinds of water samples, including paddy field water, river water, and pond water, were collected from a rural area (Louxing district, Loudi city, China). The presence of FM in these samples was detected using the standard addition method under optimized conditions. Initially, the water samples were subjected to filtration with a 0.45 μm filter membrane to eliminate larger-particle impurities. Subsequently, the samples were adjusted in an acetic acid buffer solution (0.1 M, pH = 7.0). FM standard stock solution was added to the buffered water samples to prepare spiked solutions at concentrations of 5.0, 50, and 200 nM. For the MIP analysis, the developed MIP/Ti_3_C_2_T_x_/GCE was immersed in 10 mL of each spiked sample solution for 30 min. After this incubation period, the electrode was washed with distilled water and then analyzed using the DPV to determine the concentration of FM.

## 3. Results and Discussion

### 3.1. Characterization of Ti_3_C_2_T_x_/GCE and MIP/Ti_3_C_2_T_x_/GCE

**Figure 2:**
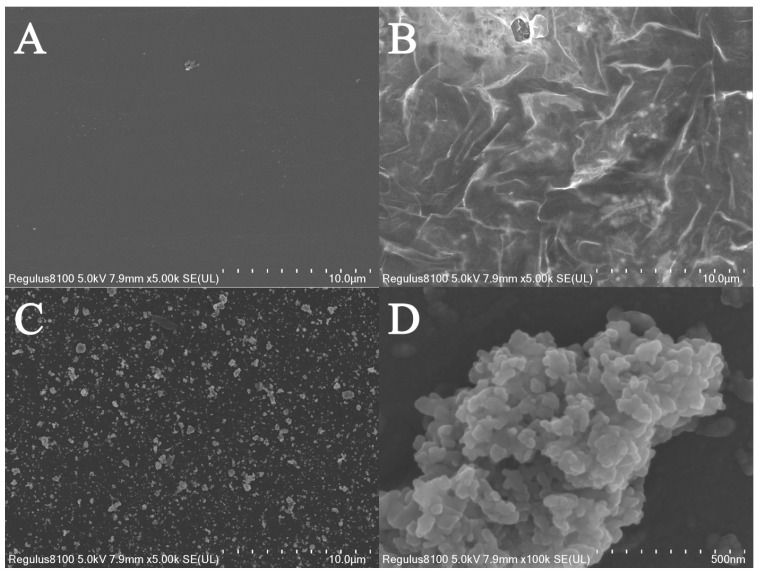
The SEM images of (**A**) bare GCE, (**B**) Ti_3_C_2_T_x_/GCE, and (**C**) MIP/Ti_3_C_2_T_x_/GCE, and (**D**) the enlarged SEM image of MIP/Ti_3_C_2_T_x_/GCE.

The morphology of each surface of the different electrodes was investigated using a scanning electron microscope. As shown in Figure 2A, the bare electrode exhibited a smooth surface. In contrast, after modification with Ti_3_C_2_T_x_, the Ti_3_C_2_T_x_-modified electrode displayed a wrinkled morphology (Figure 2B), which is consistent with previous reports [24]. This wrinkled structure is advantageous as it provides a larger specific surface area and facilitates electron transfer [22,24]. Following the modification with molecularly imprinted polymers, the electrode surface was covered with numerous irregular polymeric aggregates (Figure 2C), which exhibited a distinct morphology compared to that of the Ti_3_C_2_T_x_-modified electrode. In particular, the locally enlarged image (Figure 2D) highlighted that the surface of these irregular polymeric aggregates had a three-dimensional structure and roughness. This characteristic is significant as it creates more binding sites for template molecules, thereby enhancing the efficiency of molecular recognition.

### 3.2. Ti_3_C_2_T_x_/GCE Preparation Optimization

**Figure 3:**
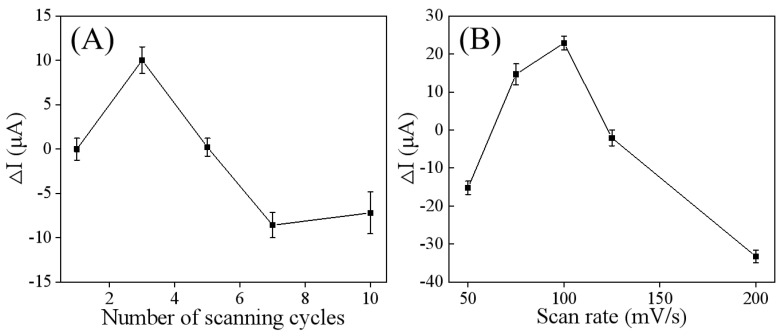
Optimization of factors affecting the electrodeposition performance of the Ti_3_C_2_T_x_/GCE: the number of scanning cycles (**A**) and scan rate (**B**). ΔI represents DPV peak current change (ΔI = I − I_0_) before and after electrodeposition of Ti_3_C_2_T_x_.

Considering that the thickness and uniformity of the modified material on the electrode affect the electrical signal intensity and analytical sensitivity at the electrode interface, various factors impacting the preparation of the Ti_3_C_2_T_x_/GCE were optimized. Specifically, the number of CV scanning cycles and the scan rate were adjusted during the CV deposition process. As shown in Figure 3A, the ΔI value of the DPV peak current increased with the number of scanning cycles, but it began to decrease after three cycles. This indicates that three cycles is optimal for achieving the desired electrochemical properties of Ti_3_C_2_T_x_/GCE. Fewer scan cycles led to uneven or defective deposition of Ti_3_C_2_T_x_, resulting in weak electron transfer capability. Conversely, too many scanning cycles caused the deposition to become overly thick, which reduced the electrode’s electron transferability and conductivity. The scan rate is another crucial parameter influencing the electrodeposition of Ti_3_C_2_T_x_, as it significantly affects deposition quality and overall electrode performance. As show in Figure 3B, within the scan rate range of 50–200 mV/s, the ΔI value of the DPV peak current increased with the scan rate, but decreased when the scan rate exceeded 100 mV/s. This phenomenon is consistent with the previous findings that a lower scan rate can achieve better deposition quality and more uniform surface morphology, although it requires a longer deposition time [24]. In contrast, a higher scan rate can accelerate the deposition process but may lead to uneven or defective deposition.

### 3.3. Electrochemical Investigations

**Figure 4:**
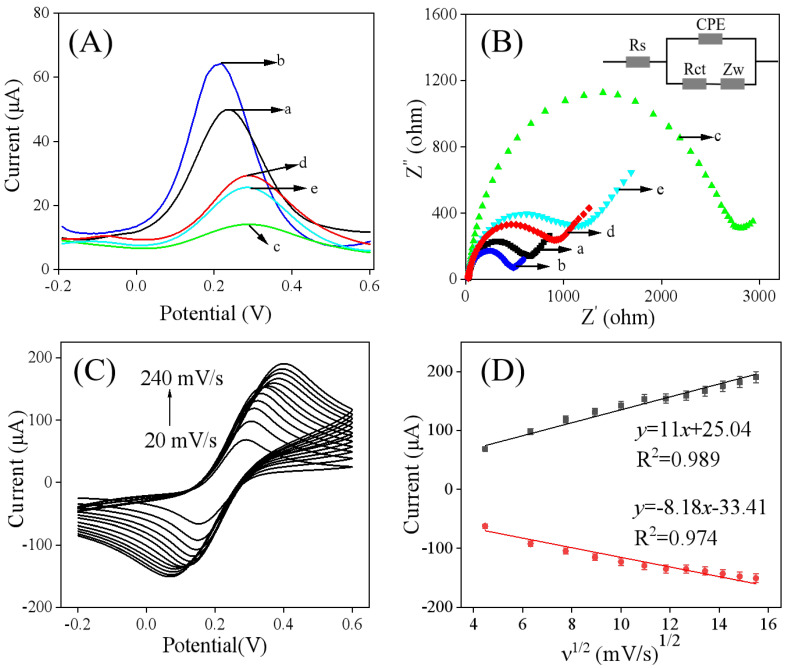
(**A**) DPV and (**B**) curves of different electrodes for EIS conducted in a 0.1 M KCl supporting electrolyte containing 5.0 mM of [Fe(CN)_6_]^3−/4−^, bare GCE (a), and Ti_3_C_2_T_x_/GCE (b), MIP/Ti_3_C_2_T_x_/GCE before (c) and after (d) removing of FM, and MIP/TTi_3_C_2_T_x_/GCE after incubation in 50 nM FM (e). (Inset is the Randles circuit model for the modified electrodes. Rs, electrolyte solution resistance; Rct, interfacial electron transfer resistance element; Zw, Warburg impedance resulting from ion diffusion; CPE, constant phase angle element.) (**C**) CV curves of MIP/Ti_3_C_2_T_x_/GCE at scan rates ranging from 20 to 240 mV/s, and (**D**) linear relationship between ν^1/2^ and the peak current.

DPV was employed to characterize the electrochemical performance of the modified electrodes. As shown in Figure 4A, the DPV peak current response of the Ti_3_C_2_T_x_/GCE (curve b) was greater than that of the bare GCE (curve a). This enhancement can be attributed to the high surface area and excellent electrical conductivity of Ti_3_C_2_T_x_, indicating successful modification of the electrode. Furthermore, after the electropolymerization of catechol, the peak current decreased significantly (curve c), suggesting the formation of an insulting imprinted membrane that hinders electron transfer. Following the removal of FM (the template) molecules (curve d), the peak current increased noticeably, indicating successful template removal and the formation of cavities in the polymer film. This increase allows the redox probe to diffuse more readily to the electrode surface [18,19]. In particular, after incubating the MIP/Ti_3_C_2_T_x_/GCE in 50 nM FM, the peak current decreased again (curve e), demonstrating that FM can rebind to the imprinted cavities in the polymer network, thereby obstructing the access of the [Fe(CN)_6_]^3−/4−^ probe.

Electrochemical impedance spectroscopy (EIS) serves as another effective technique for evaluating the electrochemical properties of electrode surfaces [25,26]. Based on the Nyquist plots presented in Figure 4B, the diameter of the semicircle region at high frequencies is proportional to the value of charge transfer resistance (Rct). The experimental data were simulated using a Randles equivalent circuit. As shown in Figure 4B and Table 1, the Rct value of Ti_3_C_2_T_x_/GCE (curve b) significantly decreased compared to that of GCE (curve a), which indicates the capacity of Ti_3_C_2_T_x_ to enhance electron transfer at the electrode surface. Following the electropolymerization of catechol, an increase in the Rct value was observed (curve c). With the removal of FM, there was a subsequent decrease in Rct (curve d), suggesting that the imprinted cavities were retained on the molecularly imprinted polymer’s surface. It is noteworthy that when the MIP/Ti_3_C_2_T_x_/GCE was exposed to an FM solution, the DPV peak current (Figure 4A, curve e) decreased, while the AC electrochemical impedance increased (Figure 4B, curve e). This change was attributed to the rebinding of FM, which obstructed the access of the [Fe(CN)_6_]^3−/4−^ probe, thereby indicating the potential for developing a selective detection method for FM molecules by utilizing selective rebinding to inhibit the probe’s access.

Cyclic voltammetry (CV) curves recorded at different scan rates represents the typical method of investigating the electroactive surface area (*A_e_*) of MIP/Ti_3_C_2_T_x_/GCE [27,28]. As shown in Figure 4C,D, both the anodic and cathodic peak currents increased with increasing scan rate (ν) and exhibited a linear correlation with the square root of the scan rate, indicating a diffusion-controlled electrochemical reaction system. According to previous studies [27,28], the electroactive surface area (*A_e_*) of MIP/Ti_3_C_2_T_x_/GCE can be evaluated based on the formula below:

(1) Ip=2.687×105n23AeD12cν12

where *n* refers to the electron transfer number (*n* = 1), *D* refers to the diffusion coefficient (cm s^−1^, *D* = 7.6×10^−6^ cm^2^ s^−1^), and c refers to the concentration of the probe (mol cm^−3^). According to the slope of Ip versus *ν*^1/2^, *A_e_* of MIP/Ti_3_C_2_T_x_/GCE was determined to be 0.29 cm^2^, which is larger than that of bare GCE (*A_e_* = 0.071 cm^2^) [29], thereby enhancing the sensitivity of the sensor.

### 3.4. Preparation and Optimization of Parameters for the Developed MIP/Ti_3_C_2_T_x_/GCE Sensor

**Figure 5:**
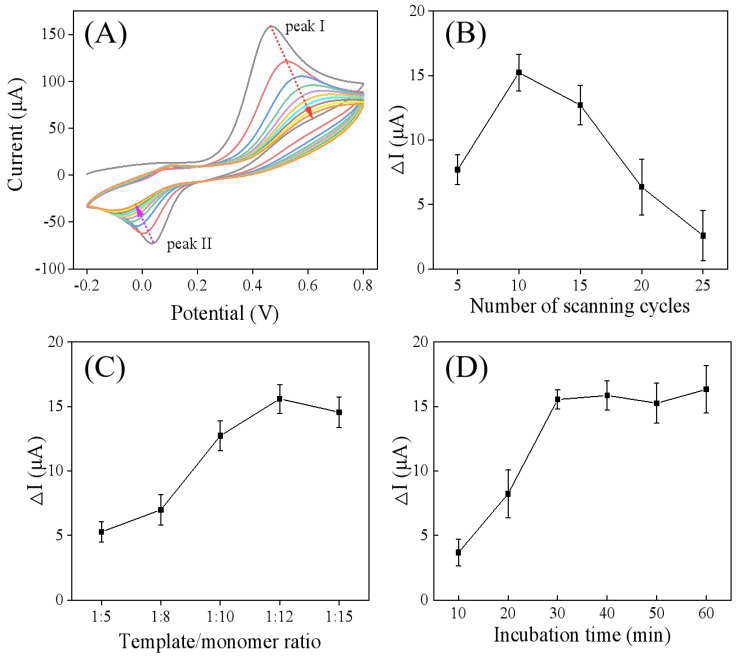
(**A**) Cyclic voltammograms corresponding the electrodeposition of catechol–FM on Ti_3_C_2_T_x_/GCE. Optimization of the experimental parameters for MIP/Ti_3_C_2_T_x_/GCE preparation: (**B**) the number of CV scanning cycles, (**C**) template-to-monomer ratios, and (**D**) incubation time.

The molecularly imprinted electrode (MIP/Ti_3_C_2_T_x_/GCE) was prepared with the electropolymerization method through CV scanning in the presence of catechol and FM. As illustrated in Figure 5A, the CV curve of catechol displayed an anodic peak (I) and a corresponding cathodic peak (II), which were attributed to the oxidation and reduction of catechol to o-benzoquinone, respectively [30]. With an increasing number of cycles, a decrease in peak currents and an increase in peak potential separation (ΔEp) were observed, indicating the formation and growth of catechol polymers on the electrode. During the electropolymerization process, it was speculated that FM could be entrapped in the cross-linked catechol polymer network through hydrogen bond interactions, where catechol acts as a hydrogen bond donor, and FM, with its nitrogen-containing heterocyclic ring, serves as a hydrogen bond acceptor. This speculation was verified by the DPV and impedance spectroscopy results. As depicted in Figure 4A,B, the MIP/Ti_3_C_2_T_x_/GCE exhibited an increase in DPV peak current (Figure 4A, curve d) and a decrease in electrochemical impedance (Figure 4B, curve d) compared to the electrode obtained after the electropolymerization step (Figure 4B,C, curve c), confirming the formation of an FM molecularly imprinted film on the Ti_3_C_2_T_x_/GCE surface.

The thickness of the molecularly imprinted membrane, which influences both the diffusion rate of FM and the response signal of the imprinted sensor, can be regulated by adjusting the number of CV scanning cycles. As shown in Figure 5B, the ΔI value of the DPV peak current increased with the number of scanning cycles up to 10 cycles, followed by an immediate decrease. This reduction in current response suggests a decrease in imprinting capacity, as the increased thickness of the imprinted membrane restricts access to the molecular recognition sites and inhibits the diffusion of the redox probe to the electrode surface [20]. Consequently, 10 cycles was selected as the optimal number for subsequent electropolymerization.

The impact of varying template-to-monomer ratios on sensor performance was investigated with varying template/monomer ratios (1:5, 1:8, 1:10, 1:12, 1:15), where the concentration of FM was fixed at 1.0 mM. As shown in Figure 5C, when the ratio was 1:12, the ΔI value of the DPV peak current was the largest when the ratio was 1:12. Both lower and higher ratios led to a decrease in the ΔI value. This is because insufficient functional monomer concentration leads to the generation of fewer imprinted recognition sites, while an excessively high concentration prevents hydrogen-bonding-recognition interactions from occurring during electropolymerization [21]. Therefore, the optimal template-to-monomer ratio was determined to be 1:12.

Given that the incorporation of the template into the polymer film is significantly influenced by incubation time, this parameter was optimized. As shown in Figure 5D, with increasing incubation time, the ΔI value of the DPV peak current initially increased and stabilized approximately after 30 min, signifying that the adsorption capacity had reached saturation. Consequently, 30 min was designated as the optimal incubation time for subsequent experiments.

### 3.5. Analytical Performance of MIP/Ti_3_C_2_T_x_/GCE for Quantification of Fenclorim

**Figure 6:**
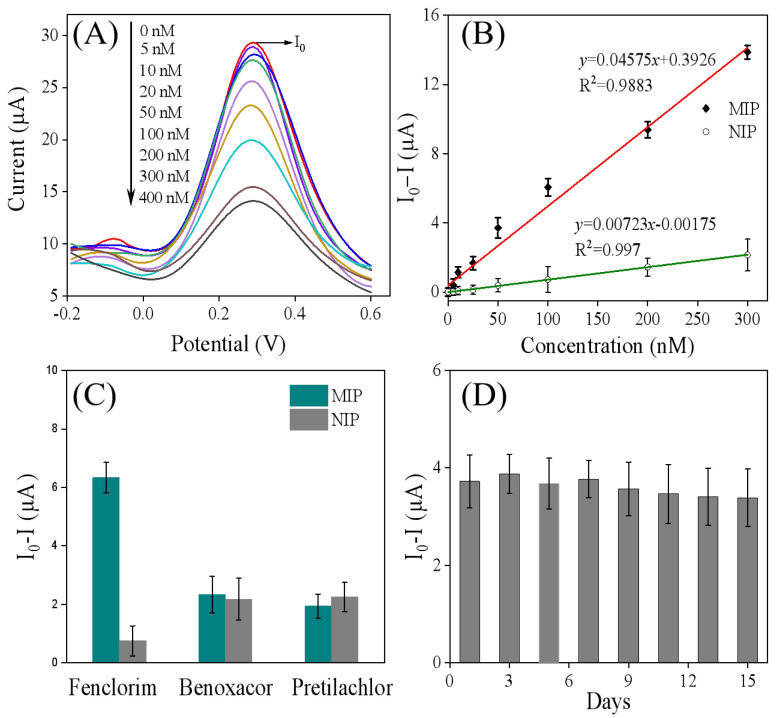
(**A**) The DPV curve generated after the MIP/Ti_3_C_2_T_x_/GCE was incubated in different concentrations of FM: 0, 5, 10, 25, 50, 100, 200, 300, and 400 nM. (**B**) The linear relationship between FM concentration and the changes in DPV peak current for MIP/Ti_3_C_2_T_x_/GCE (red line) and NIP/Ti_3_C_2_T_x_/GCE (green line), where I_0_ represents the DPV value of MIP/Ti_3_C_2_T_x_/GCE incubated without FM, and I represents the DPV value of MIP/Ti_3_C_2_T_x_/GCE incubated with varying FM concentrations. (**C**) The DPV current responses for FM and other structural analogs on the MIP sensor and NIP sensor. Here, I_0_ represents the DPV value of electrodes incubated without FM, and I represents the DPV value after incubation with either 100 nM FM or 1.0 mM of other structural analogs. (**D**) The DPV peak current responses of MIP/Ti_3_C_2_T_x_/GCE after incubation with 50 nM of FM over various durations (1, 3, 5, 7, 9, 11, 13, and 15 days). In this case, I_0_ indicates the DPV value of electrodes incubated without FM, while I represents the DPV value after incubation with 50 nM FM.

To verify the applicability of the developed MIP sensor for quantitative analysis of fenclorim (FM), the response of this electrode to varying concentrations of FM was investigated via DPV measurements under optimal operating conditions. As shown in Figure 6A,B, the DPV responses of MIP/Ti_3_C_2_T_x_/GCE decreased upon increasing the FM concentration. This phenomenon can be attributed to the occupation of imprinted cavities in MIP/Ti_3_C_2_T_x_/GCE by FM molecules. Notably, when the FM concentration exceeded 300 nM, the rate of decrease in the DPV signal slowed, suggesting that adsorption equilibrium may be approaching. As depicted in Figure 6B, a linear relationship was observed between the peak current difference and FM concentration in the range of 5.0–300 nM, with a limit of detection (LOD, S/N = 3) of 1.56 nM [31,32]. The linear regression equation was expressed as follows: ΔI(μA) = 0.04575c (nM) + 0.3926 (R^2^ = 0.988). In contrast, the non-imprinted polymer (NIP) electrode exhibited a linear concentration range of 5–300 nM for FM, with an LOD of 3.27 nM, and its corresponding linear regression equation was ΔI(μA) = 0.00723c (nM) − 0.00175 (R^2^ = 0.997). However, the response generated by the NIP sensor is likely due to non-specific adsorption of the target compound on the electrode surface [20,21,26]. The imprinting factor (defined as the ratio of sensitivity for MIP to that of NIP) was calculated to be 6.3, indicating that the MIP based sensor possesses excellent selective-recognition capability for the template molecule. Notably, as shown in Table 2, the MIP/Ti_3_C_2_T_x_/GCE sensor prepared in this study exhibits a comparable detection limit to the commonly used high-performance liquid chromatography–diode array detector (HPLC-DAD) methods reported previously, demonstrating its promising application potential for FM analysis.

### 3.6. Selectivity and Stability

To further investigate the selectivity of the molecularly imprinted sensor, it was evaluated in solutions containing FM (100 nM) along with interfering substances at 10-fold higher concentrations, such as pretilachlor or benoxacor. Pretilachlor is a chloroacetamide herbicide that provides pre-emergence control of annual grassy weeds and some broadleaf weeds in rice [11]. Benoxacor and FM are commonly used as safeners and are often combined with dichloroacetamide herbicides (with the safener additive accounting for less than 10%) because they can alleviate the phytotoxicity of herbicides on crops [3,33]. As shown in Figure 6C, the NIP electrode exhibited almost identical responses to FM and other interfering substances. This is because there are no imprinting cavities in NIP that match FM, resulting in a lack of selectivity. In contrast, the MIP electrode showed a high current response only to FM, while its responses to several interfering substances were low; furthermore, there was almost no significant difference in current responses to different interferents. These results indicate that the prepared MIP electrode not only has a better current response but also maintains high selectivity and specific recognition ability for FM. This can be attributed to the fact that FM can occupy the imprinted cavities through hydrogen bonding, leading to an obvious change in current. The stability of the molecularly imprinted sensor was also evaluated over 15 days under laboratory conditions. As shown in Figure 6D, the DPV peak current responses decreased by less than 10%, demonstrating that the fabricated sensors have appropriate stability.

### 3.7. Real-Sample Analysis

The constructed MIP/Ti_3_C_2_T_x_/GCE was applied to detect FM in real water samples using the standard addition method to evaluate its practical application performance. Typically, the newly developed MIP/Ti_3_C_2_T_x_/GCE was immersed in FM-spiked solution for 30 min, rinsed with distilled water, and measured with the DPV method to collect the peak currents of the [Fe (CN)_6_]^3−/4−^ probe. As shown in Table 3, the recovery of FM ranged from 93.07% to 109.28%, with relative standard deviations (RSD) all below 5.0%. These results demonstrate that the constructed MIP sensor can effectively and accurately determine FM in real samples.

## 4. Conclusions

This study focused on developing an electrochemical sensor for FM detection in water samples using molecularly imprinted polymers. The incorporation of Ti_3_C_2_T_x_ through electrodeposition significantly enhanced the conductivity of the sensing system. The sensor demonstrated a linear response for FM concentrations ranging from 5 to 300 nM, achieving a detection limit of 1.56 nM. This detection limit is comparable to that of high-performance liquid chromatography with a diode array detector (HPLC-DAD). The sensor’s effectiveness was validated with real water samples, showing acceptable recovery rates. In conclusion, the study successfully established a simple, sensitive, and effective electrochemical sensor based on molecularly imprinted polymers for detecting FM in water samples.

## Figures and Tables

**Table 1:** Simulated experimental data derived from equivalent electrical circuit molding.

Electrode	Rs (ohm·cm^2^)	Rct (ohm·cm^2^)	Warburg	CPE	
			Y_0_ (S·s^5^·cm^−2^)	Y_0_ (S·s^n^·cm^2^)	*n*
**Bare GCE**	2.08	39.68	0.007	1.904 × 10^−5^	0.87
**Ti_3_C_2_T_x_/GCE**	1.46	30.54	0.015	1.772 × 10^−5^	0.80
**MIP without elution**	1.64	188.20	0.015	2.074 × 10^−5^	0.89
**MIP/Ti_3_C_2_T_x_/GCE**	1.78	56.71	0.003	2.112 × 10^−5^	0.87
**MIP incubated in 50 nM FM**	1.89	69.58	0.005	1.342 × 10^−5^	0.85

**Table 2:** Comparison of the developed method for the determination of FM with other methods reported in the literature.

Method	Linear Range	LOD	Reference
HPLC-DAD	-	<0.02 μg g^−1^ (88.89 nM)	[12]
Solid-phase extraction tandem GC/MS	-	2.00 ng/L (8.89 pM)	[13]
MIP-Ti_3_C_2_T_x_/GCE	5.0–300 nM	1.56 nM	This paper

**Table 3:** Determination and recovery of FM in the real water samples on MIP/Ti_3_C_2_T_x_/GCE (*n* = 3).

Sample	Add (nM)	Found (nM)	Recovery (%)	RSD (%)
Paddy field water	5	4.93	93.07	4.5
	50	52.36	104.71	3.5
	200	198.89	99.45	2.4
Pond water	5	5.46	109.28	4.9
	50	50.25	100.51	2.5
	200	203.76	101.88	3.8
River water	5	50.41	100.82	4.2
	50	48.33	96.66	4.0
	200	205.69	102.84	2.9

## Data Availability

The original contributions presented in this study are included in the article; further inquiries can be directed to the corresponding author.
